# Intramuscular schwannoma of the musculocutaneous nerve: An uncommon clinical presentation

**DOI:** 10.3892/etm.2013.1084

**Published:** 2013-04-29

**Authors:** JUN NISHIO, TAKAYUKI UEKI, MASATOSHI NAITO

**Affiliations:** Department of Orthopaedic Surgery, Faculty of Medicine, Fukuoka University, Fukuoka 814-0180, Japan

**Keywords:** coracobrachialis muscle, enucleation, intramuscular, magnetic resonance imaging, musculocutaneous nerve, schwannoma

## Abstract

A schwannoma is a benign peripheral nerve sheath tumor composed exclusively of Schwann cells. A major-nerve schwannoma with an intramuscular location is an extremely rare condition. We present a rare case of intramuscular schwannoma originating from the musculocutaneous nerve in a 71-year-old female. The patient presented with a 7-month history of a slowly growing, painless mass in the medial aspect of the proximal upper arm. Magnetic resonance imaging revealed an oval-shaped intramuscular soft tissue mass with iso-signal intensity relative to skeletal muscle on T1-weighted images and high signal intensity on T2-weighted images. A rim of fat surrounding the mass, suggesting the split-fat sign, was also observed. The tumor was completely enucleated using an intracapsular technique. Histological examination confirmed the diagnosis of schwannoma consisting of Antoni A and B areas. There was no immediate neurological deficit following surgery. The patient had no evidence of local recurrence and no neurological deficit at final follow-up. To the best of our knowledge, this is the first report of musculocutaneous nerve schwannoma within the coracobrachialis muscle.

## Introduction

A schwannoma is a benign encapsulated nerve sheath tumor arising from Schwann cells. It occurs predominantly in middle-aged adults with no gender predilection. Patients typically present with a slowly growing, painless mass in the head, neck and flexor surfaces of the upper and lower extremities. An intramuscular schwannoma is rare and its clinical behavior may be different from that of a schwannoma occurring in other locations (1,2). We present an unusual case of an intramuscular schwannoma originating from the musculocutaneous nerve in an elderly female. To the best of our knowledge, this is the first description of a musculocutaneous nerve schwannoma within the coracobrachialis muscle. Written informed consent for publication was obtained from the patient.

## Case report

**Figure 1.**
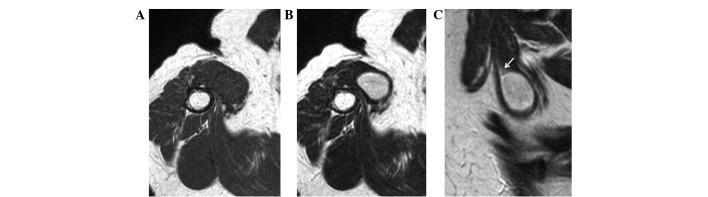
On magnetic resonance imaging, the mass has (A) iso-signal intensity relative to adjacent muscle on the T1-weighted image and (B) high signal intensity on the T2-weighted image. (C) The sagittal T2-weighted image shows the mass with surrounding fat (arrow), representing the split-fat sign.

A 71-year-old female was referred to Fukuoka University (Fukuoka, Japan) with a 7-month history of a slowly growing, painless mass in the medial aspect of the right proximal upper arm. Physical examination revealed an elastic-hard, poorly mobile, non-tender mass. Neurovascular examinations, including Tinel’s sign, were normal. Laboratory data were within normal limits. The patient’s past medical history was unremarkable. Magnetic resonance imaging (MRI) demonstrated an oval-shaped soft tissue mass in the right coracobrachialis muscle, measuring 3.0×2.0×2.0 cm. The mass presented homogeneous iso-signal intensity relative to skeletal muscle on T1-weighted images (Fig. 1A) and high signal intensity on T2-weighted images (Fig. 1B). A rim of fat surrounding the mass was also observed (Fig. 1C). Based on these findings, a preoperative diagnosis of a benign neurogenic tumor, including intramuscular schwannoma was made.

**Figure 2.**
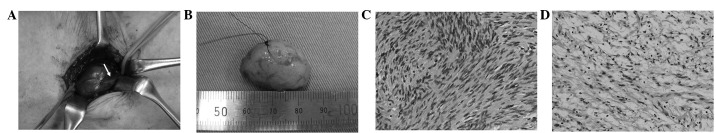
(A) Intraoperative image of the tumor and the musculocutaneous nerve (arrow). (B) Gross appearance of the tumor. Photomicrographs reveal (C) more cellular Antoni A areas and (D) loosely textured Antoni B areas.

Surgery was performed under general anesthesia. First, the tumor and the proximal and distal portions of the affected nerve were exposed (Fig. 2A). A longitudinal incision was carefully made in the epineurium far away from the fascicles. The epineurial layers were gently peeled away until the shiny surface of the tumor was exposed. The entire tumor mass was shelled out in one piece without damage to the fascicles. Intraoperative findings were consistent with the diagnosis of schwannoma. Grossly, the smooth-surfaced tumor was yellow-whitish (Fig. 2B). Microscopically, the tumor demonstrated a proliferation of spindle-shaped cells arranged in short bundles or interlacing fascicles in Antoni A areas (Fig. 2C). Edematous, hypocellular areas known as Antoni B were also observed (Fig. 2D). Neither nuclear atypia nor mitotic figures were observed. These features confirmed the diagnosis of a schwannoma.

There was no immediate neurological deficit following surgery. At six months of follow-up, the patient had no evidence of recurrence and no neurological deficit.

## Discussion

The musculocutaneous nerve arises from the lateral cord of the brachial plexus. It penetrates the coracobrachialis muscle at the level of the tendon of the *latissimus dorsi* muscle and passes obliquely between the biceps brachii muscle and the brachialis muscle to continue into the forearm as the lateral antebrachial cutaneous nerve (3,4). Its initial branches are motor in function and the remaining fibers are sensory in function at the cubital fossa. Although few cases of schwannoma originating from the musculocutaneous nerve have been documented (2,5), there is, to the best of our knowledge, no literature describing a musculocutaneous nerve schwannoma within the coracobrachialis muscle.

The histological hallmark of a schwannoma is the pattern of alternating Antoni A and B areas, as demonstrated in the present case. The relative amounts of these two components vary and may blend imperceptibly or change abruptly. Antoni A tissue is highly cellular and demonstrates nuclear palisading and associated Verocay bodies. Antoni B tissue is less cellular and lacks distinctive architectural features. A number of schwannomas have thick-walled vessels with fibrinoid and hyaline changes in the vessel walls. When examined by immunohistochemistry, schwannomas typically show diffuse, strong expression of S-100 protein and abundant pericellular collagen type IV (6). Unlike neurofibroma, neurofilament protein staining is usually limited to entrapped axons at the periphery of the tumor.

Intramuscular schwannomas are rare (5); they originate from a small nerve branch within the muscle. The clinical features are different from those of schwannomas occurring in other locations (1,2). In intramuscular schwannomas, neurological symptoms or signs, including pain, Tinel’s sign, sensory disturbance or motor weakness, are few. It may therefore be difficult to identify the neurogenic origin based on physical examination. Using MRI, the majority of lesions demonstrate iso- or low signal intensity on T1-weighted images and high signal intensity on T2-weighted images, as shown in the present case. Post-contrast images show marked central enhancement (1). In the present case, the origin of the tumor was not a small motor branch but a trunk of the musculocutaneous nerve. Despite its rare occurrence, it is important to be aware of the possible existence of a major nerve trunk schwannoma in the coracobrachialis muscle.

Enucleation is a standard surgical procedure for schwannomas. However, certain schwannomas are not easily enucleated and enucleation may result in iatrogenic nerve injury, even with atraumatic procedures. Donner *et al* (7) recommended extracapsular enucleation with good results; however, the procedure is likely to damage the fascicles in the capsular layer during dissection. Previously, intracapsular enucleation has been performed to minimize the risk of nerve injury (8–10). Date *et al* (9) reported that neurological deficit following enucleation is significantly lower using the intracapsular compared with the extracapsular technique. The authors mentioned that en bloc resection should not be performed since the main purpose of schwannoma surgery is the relief of symptoms. In the present case, gentle dissection along the plane of the tumor capsule from the epineurial layers allowed the tumor to be shelled out in one piece without disturbing the fascicles.

In summary, we have reported the first case of an intramuscular schwannoma originating from the musculocutaneous nerve. Although rare, schwannomas should be included in the differential diagnosis of a well-defined, oval-shaped soft tissue mass arising within the coracobrachialis muscle.
